# Sengi exceptionalism

**DOI:** 10.3389/fnins.2014.00281

**Published:** 2014-09-04

**Authors:** Kathleen S. Rockland

**Affiliations:** Anatomy and Neurobiology, Boston University School MedicineBoston, MA, USA

**Keywords:** brain size, modularity, enlarged hippocampus, structure-function relationships, habitat mapping

The recent report of Slomianka et al. (2013) starts from the fact that the hippocampus is disproportionately large in the eastern rock sengi. The sengi, or elephant shrew, is a small mammal, native to southern Africa, previously grouped with insectivores but now considered to form a separate taxonomic order (*Macroscelidae*). A disproportionately large hippocampus occurs in a number of other species, such as food-storing birds or hummingbirds, and has been correlated with behaviors involving an extra demand for spatial learning and memory (Frahm and Zilles, 1994; Biegler et al., 2001; Ward et al., 2012). Such behaviors can be learned (i.e., as in London taxi drivers) or species specific. In this particular case, the authors do not venture to speculate as to why the sengi has an enlarged hippocampus; and, this is perhaps a wise choice, given the uncertainties and controversies surrounding comparative studies of brain size (e.g., Healy and Rowe, 2007). For example, elephants, close relatives to elephant shrews, do not have an “unduly enlarged” hippocampus, despite their strong reliance on long-term spatial memory (Patzke et al., 2013a). Cetaceans have relatively small hippocampi, although the possible behavioral correlations with complex cognition remain under debate (Patzke et al., 2013b).

The authors emphasize three main findings. One is that the large and well-differentiated sengi hippocampus is not associated with corresponding hypertrophy of cortical and subcortical areas that are the main input sources and target outputs for the hippocampus. That is, the literature does not support commensurate size changes in the wider interconnected hippocampal network, as measured for entorhinal and septal structures (Stephan, 1983). As the authors remark, the unique change in hippocampal volume seems to point to intrinsic hippocampal processing, and not network processing, as the operative selective advantage.

A second emphasis of the study is a detailed cytoarchitectural and quantitative analysis of the major hippocampal subfields. This consists of determining total cell numbers (Table 2; *n* = 8 individuals), and then deriving the degree of convergence or divergence between the subfields, on the basis of the respective cell numbers. From comparison between the eastern rock sengi and nine other species, the authors find (Table 3) that the degree of convergence of CA1 pyramidal cells onto subicular target neurons “by far exceeds” the values observed in the other species.

A further correspondence analysis of total cell numbers and quantitative populational relations supports several observations with interesting implications for taxonomy and comparative microcircuitry. That is, both the sengis and rhesus monkeys have a high degree of convergence (many-to-fewer) from granule cells of the dentate gyrus to CA3 pyramids; the cellular divergence (fewer-to-many) from CA3 to CA1 pyramids is similar for the sengis, primates, dogs, and domestic pigs and larger in those species than for rats, mice, and tree shrews. An incidental but important consequence of this range, as the authors further point out, is that generalized models of hippocampal function need to be robust (up to fivefold differences in ratios) to large quantitative differences in connectional convergence and divergence among the several interconnected hippocampal subfields.

In parallel to the quantitative analysis of pyramidal cell populations, the authors undertake a quantitative analysis of neurochemically distinct subgroups. This follows on a broader cross-species survey (Slomianka et al., 2011) of significant histological, neurochemical, and connectivity differences between deep and superficial pyramidal neurons in CA1 and CA3. In the present report, among other observations, the authors find that calbindin is not expressed by superficial pyramidal cells in CA1 of the sengi, a trait shared with guinea pigs and rabbits but contrasting with mice, fox, and primates. Age-, activity-, or hormone-dependent factors are mentioned as possible mechanisms underlying these species-specific expression patterns. The distributions of parvalbumin and somatostatin interneurons resemble those described in other species.

The third main result addresses how a very large hippocampus impacts on adult hippocampal neurogenesis (AHN), and in particular sets out to test previous observations in mice that a large habitat can be associated with either a large dentate gyrus and low level of AHN or a smaller dentate gyrus and a high level of AHN (Amrein et al., 2004). The answer, not surprisingly, turns out to be complicated. Despite the exceptionally high number of granule cells in the sengi dentate gyrus, normalized numbers of proliferating cells and doublecortin-positive differentiating cells were found to fall within the overall range of murid rodents, although the greater similarity was with Muridae collected from a European habitat. A unique influence of habitat is not immediately obvious since (1) AHN did not differ between male and female sengis, despite the smaller absolute size of the home range for females, and (2) AHN did differ between sengi and, with one exception, mouse species sharing the same (African) habitat. (See Cavegn et al., 2013 for a longer discussion of the involvement of AHD specifically in fast behavioral changes and how various subtle aspects of the environment might interact with rates of AHD).

In summary, this paper is a careful quantitative analysis that, given the interesting phylogenetic status of the sengi, fills a conspicuous gap in the database of hippocampal parameters (Kempermann, 2012). More data, of course, are needed. The authors themselves mention the need for more developmental time points for the data on AHN. One might also ideally want connectivity data in relation to hippocampal long-axis specializations (e.g., Poppenk et al., 2013), and more detailed data at the level of axonal projections, synaptic numbers, and dendritic arbors. Impressively, however, the paper also points toward an eventual integration of several major issues. Modularity: why is the sengi hippocampus enlarged in apparent isolation from structures closely interconnected with the hippocampus? What is the role of species vs. habitat (as, nature/nurture) in adult neurogenesis and hippocampal subfield composition?

Finally, the results directly relate to major conceptual issues, such as the need to avoid simplistic structure-function correlations (Healy and Rowe, 2007; Finlay and Workman, 2013; Uddin, 2013; Anderson and Finlay, 2014), and the need to address complicated interactions, both within the brain and between the brain, the outside environment, and genetic, hormonal, and other major body systems. The sengi, with its enlarged hippocampus, offers an excellent non-sensory model system to probe these issues, potentially on a par with the rodent barrel cortex for the sensory domain.

## Conflict of interest statement

The author declares that the research was conducted in the absence of any commercial or financial relationships that could be construed as a potential conflict of interest.
